# Nature and Art in Curing Diseases: A Guide for Young Physicians

**Published:** 1836-01

**Authors:** 


Art. VI.—*Natur und Kunst in heilung der Krankheiten: ein Leitfaden für angehende Aerzte.* Von Dr. Johann Jacob Günther, Königl. Preussischem und herzogl. Nassauischem Medicinalrathe, u. s. f. *Frankfurt am Main.* 1834.

*Nature and Art in curing Diseases: a Guide for young Physicians.* By Dr. J. J. Günther, *Frankfort on the Maine.* 1834. Small 8vo. Pp. 229.

This little work is a short view of the theory of medicine, considered under two heads; the one being an account of the healing powers of nature, the other of art. In treating of the vis medicatrix naturæ, our author subdivides it into two kinds, each of which is treated in a separate section; the one, where the salutary influence is quite independent of the patient's will or feelings, as when a fit of apoplexy is warded off or relieved by epistaxis or hemorrhoids; the other where the cure depends on the imagination or strong will of the sick man. Kant, the metaphysician, wrote a treatise on this last point, entitled, "On the power of the mind in becoming master of its morbid sensations, by the force of resolution alone;" in which he relates his own case, as follows. He was subject to a cough, which tormented him at night, caused by the irritation of the inspired air upon the larynx; and this he quelled by strongly directing his attention to some other object. The expiration of the air was thus stopped, and he felt the rush of blood into his face, which caused a flow of saliva; and a new irritation being thus substituted for the former one, the moisture was swallowed, and the cough ceased. "This operation of the mind (adds Kant) requires firm resolution in a very high degree, but, on that very account, is the more beneficial. The following passage, taken from the section on the unassisted powers of nature, may serve to show the good sense of Dr. Günther, as well as the even tenor of his style. After speaking of the instinct which leads animals to avoid dangerous things and places, to clear away unwholesome dirt, to lick their wounds, and even to seek out herbs against disease, he adds:

"Nor is this instinct entirely wanting in man, and it is especially beneficial in disease. Thus, those who are suffering from bilious or putrid fevers, avoid all animal food, the very sight of which causes disgust and vomiting; but, on the other hand, they have a great relish for vegetable and acid food, or rather drink. When great debility comes on, they wish for wine, which, under these circumstances, is to be preferred to all other tonics, particularly when the disease has lasted long, the voice is weak or lost, the tongue moist, and the patient not suffering from violent thirst.

When it would be pernicious, far from desiring, they dislike it. In typhus fever patients long for cold water and fresh air; neither should these, nor other things be denied them, for which they express an extreme desire, unless they are strongly contra-indicated. They are generally things which the patients relish but little in their healthy state; and, vice versâ, what pleased them most when well, is now disliked; their return to health being marked by a return to their old tastes.

"If the patient is about to experience a critical sweat, he feels a disagreeable rigor, extending over his whole body, at the slightest touch of fresh air, against which he tries to guard himself in every possible way.

"Clerc tells us that a patient labouring under general anasarca, was very desirous to have some of the St. John's bread (the Siliqua dulcis). At first he ate but little; the urine flowed more frequently; he ate again, and the quantity of urine increased from day to day, so that in a short time the swelling had entirely disappeared." P. 92-4.

The second division of the work, which treats of the value of the art of healing, is divided into two sections: one being on diseases in general, and their nature, with considerations on the difficulties lying in the way of obtaining correct experience; the second being a nearer view of the art of healing, with an attempt at forming a *catharticon* of it. The book concludes with some remarks on the formation of an Academy of Practical Medicine.

A large part of this division is taken up with an account of the symptoms of acute and chronic diseases, considered generally; and with a history of medicine, from the first dawn in Egypt, down to Broussais and Hahnemann. Dr. Günther's estimate of homœopathy is candid and judicious, his main conclusion being that homœopathy is another name for the method of treating diseases by expectation.

Dr. Günther thinks that the following are the principal obstructions to the improvement of medicine:

1. Too great an esteem for the ancients, or too great a depreciation of them.

2. Too great a preference for certain authors, and too anxious an endeavour to accommodate our observations to their system.

3. Too great a preference for particular remedies.

4. The proclivity of mankind to look upon things chiefly with reference to themselves. Thus, Baglivi complains, that physicians of a cold and melancholy temperament prescribe nothing but refrigerants, while the man of hot and acrid humours has an almost exclusive predilection for aromatic and spirituous remedies.

Dr. Günther proposes that the Medical Academy should consist of two divisions: the philosophical one to be made up of members whose duty would be to revise what has been written on medicine, and separate the wheat from the chaff; the practical one to be devoted to the investigation of disease by the treatment of cases. He thinks, and with great justice, that such an institution would do more for the improvement of physic in a few years, than ten times the period spent in the hurry of ordinary or even of hospital practice; where the wood, says our author, can often not be seen for the trees. It is to be observed, that each member of the practical department is to be confined to the treatment of some few diseases. The obvious difficulty would be to find members for the Academy. Dr. Günther's little work shows him to be a man of reading, as well as a practical physician; but it is liable to a reproach which is seldom alleged against German books, namely, that of being too short; for though the signatures are those of an octavo, the pages are very small, and the book is a pocket volume.

It is remarkable that our author, though he discusses the qualities of the urine at considerable length, and quotes numerous authors, does not mention the name of Prout. So difficult is it for the most industrious author to keep on a level with the knowledge of the age, and so slow is discovery in progressing from one country to another.

